# The metal ion theory of ageing: dietary target hazard quotients beyond radicals

**DOI:** 10.1186/1742-4933-5-3

**Published:** 2008-05-20

**Authors:** Declan P Naughton, Andrea Petróczi

**Affiliations:** 1 School of Life Sciences, Kingston University, Penrhyn Road, Kingston, London KT1 2EE, UK

## Abstract

Numerous theories of ageing exist and many are interconnected when viewed through a modern integrative biology perspective. Diet provides a link to a large number of the theories that prevail at the molecular levels. In particular, metal ions form key elements of the radical theory along with having established roles in several age-related neurodegenerative disorders. Lifetime exposure to metals has been linked to ageing by contributions to oxidative stress and neurodegenerative disorders. As many foodstuffs contain high levels and diverse profiles of metals, their cumulative effect on ageing warrants investigation. The cumulative level of concern from environmental exposure can be expressed as a dimensionless index of target hazard quotient (THQ) or for known carcinogens, the target cancer risk (TR). This paper posits that a quantifiable relationship exists between ageing and level of concern resulting from cumulated metal exposure; and that this relationship can be used to develop an ageing-related index of concern from chronic metal ion exposure. As individual differences may facilitate or moderate this cumulated exposure, the potential influence on ageing or on the development of neurodegenerative disorders should be included into the model.

## Background

For two centuries numerous theories of ageing have been proposed with variations of diversity ranging from programmed death, accumulation of mutations, antagonistic pleiotropy, radiation and radicals [1-8]. These theories are rarely mutually exclusive especially when viewed through a modern integrated biology approach. For example, the latter theory on radicals originally incorporated radiation, and has now been extended to feature redox-active metal ions, natural oxidant and anti-oxidant processes, pollutants, toxins, xenobiotics, nutrients and even pharmaceuticals. Radical damage, or oxidative stress, is a key characteristic of infection/inflammation which is also a strong candidate as a theory of ageing. Clearly, a blend of these numerous theories may be responsible in reality. The challenge may be to deconvolute the contributing mechanisms rather than to find the unifying theory. To this end, the roles of metals should be investigated beyond their contributions to oxidative stress and/or neurodegenerative disorders.

The roles of metal ions in ageing have predominated owing to their contributions to oxidative stress, neurological disorders and radiation poisoning. In addition to the established nutritional requirements and benefits which have resulted in recommended daily allowances for many metal ions, metal ions are associated with a wide range of neurological disorders giving rise to numerous reports of chelation based therapeutic agents. In addition to their roles in neurological deposit formation, metal ions are credited with activation of the immune system through their mediation of oxidative stress. The contribution of metal ions to oxidative stress has been well documented, as have the links to inflammation which is associated with the initiation and/or progression of cancer and a variety of diseases (diabetes, arthritis, cardiovascular and neurological disorders). This connectivity of roles provides the challenge when trying to ascertain the processes that underlie ageing. In addition, it is essential to disentangle the beneficial roles of individual metal ions from potential detriment afforded by multiple ions from dietary sources.

The issue of food intake has long been central to the debate on mechanisms of ageing, with extremities of the activity spectrum from calorie restriction to strenuous exercise. The paradoxical reduction in ageing afforded by both calorie restriction and exercise raises the apparent conflict of under use versus overuse. Can it be that mitochondria have a *dose response*-like optimal activity at both ends of the spectrum? It is more appropriately viewed as an issue with excess dietary intake and how the body deals with it. Obesity and related disorders are testament to the need to avoid burdening the body with processing and storage resulting from excess food intake. Beyond calorie issues, clearly identifiable through indices of obesity, less obvious dietary components such as metals are also candidates as causative agents for ageing.

It is notable that very few metals have been studied in great detail and alarming gaps in our knowledge prevail. The requirement to determine an encyclopedic knowledge of the species present for all metal ions in food and their interactions in vivo has only recently been appreciated. As for vitamins, despite a large number of studies on the beneficial and detrimental roles of metal ions, a consensus on their contributions to health and disease is still elusive. This mammoth task will, of course, take many decades. It the meantime, the roles of metals in foodstuffs and their contributions to ageing and development of age-related diseases can optimally be dissected through correlational studies as proposed herein. A key feature of these studies will be the estimation of biological age (in comparison to one's chronological age) and the combined effects of frequent ingestion of multiple metal ions as a function of the quantified level of concern in the form of target hazard quotients (THQs) arising from individual or combined metal concentrations [9,10]. In addition, THQs will require delineating as values for individual conditions as exemplified by Target Cancer Risks (TR) [9]. From this position THQ can be calculated for foodstuffs and their constituents such as metal ions, a relationship can be modeled between the biological-chronological age ration. From this relationship, a Target Ageing Quotient (TAQ) can be established to indicate the cumulative THQs' contribution to ageing.

### Presentation of the hypothesis

Two indices are typically used to quantify the level of concern of lifelong exposure to potentially toxic elements: the THQ and the TR [9]. Although the additive property of these indices would allow the assessment of the cumulative level of concern, these indices have typically focused on a single metal exposure from food contamination and the investigations have been limited to selected food items [9]. Furthermore, these indices are characterized by their environmental focus as they have been developed for assessing the level of concern for risks arising from a potentially toxic environment (i.e. arsenic content in oysters, etc.).

Lifetime exposure to metals has been linked to ageing [11]. As many foodstuffs contain high levels and diverse profiles of metals, hence the cumulative level of concern, and the contribution of individual food items should be used and their cumulative effect on ageing should be investigated. Furthermore, when investigating the effect of these potentially toxic elements, moderating effects should be taken into consideration. While dietary exposure to metals is unavoidable, the effects on health are moderated by numerous parameters such as environmental and genetic factors [12].

The metal ion theory of ageing raises numerous inter-related hypotheses, which are summarized as follows:

H1: ΣTHQ(metal) >> 1

H2: Selected dietary items (i.e. red wine and meat) and supplements (i.e. minerals) are the major contributors of high ΣTHQ(metal).

H3: Cumulated chronic metal exposure has an effect on the rate of ageing.

H4: The relationship described in H3 (and the onset of ageing related neurodegenerative diseases in some cases) are moderated by some qualitative differences between individuals or group of individuals.

### Testing the hypotheses

The hypotheses can be tested via a series of interrelated studies.

H1: ΣTHQ(metal) >> 1

The metal ion content of each dietary component of interest can be measured simultaneously using a multi-element technique such as inductively-coupled plasma-mass spectrometry (ICP-MS). From these analyses of some thirty metals, THQ values can be calculated for those with established upper safe levels (Ca, Cr, Cu, Fe, Mg, Mn, Ni, Pb, Se, Zn and V). The range of observed values can be used for the probabilistic estimation of metal content in different foodstuffs. For elements without established Tolerable Upper Intake Level (UL), THQ values will remain elusive.

*H2: Selected food items (i.e. red wine, meat) or mineral supplements are the major contributors of high ΣTHQ(metal)*.

This hypothesis can be tested by measuring the metal content in foodstuff typically ingested daily for a prolonged time. Food items will be selected based on published research and/or nationwide representative surveys. ΣTHQ(metal) can be calculated and compared to the THQ(metal) of individual food items or supplements.

H3: Relationship between ageing and ΣTHQ(metal)

A cross-sectional correlational study can be used to assess biological age and lifetime metal exposure.

The relationship between cumulated chronic metal exposure and the rate of ageing with a regression model where Y is the ratio between biological and chronological age and X = ΣTHQ(metal), which is a calculated level of concern due to exposure to metals (equation 1).

**Figure 1:**
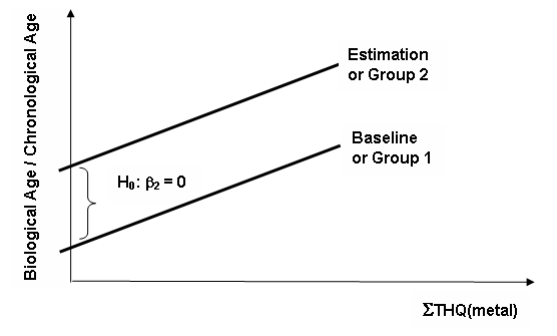
Regression models of predicting the biological age – chronological age ratio.

This regression model is depicted as a regression line in Figure 1 for illustration purpose but the regression model does not have to be limited to one predictor. In case of multiple predictors, the regression line turns into a plane or hyperplane. The biological age – chronological age ratio, which is the function of the cumulated THQ (metal) exposure is compared to a theoretical 'baseline' regression model (equation 2) where all variables are held constant except the ΣTHQ, which can reduced below the concern level (i.e. assume that the individual/population does not consume anything with significant high metal content) [13].

(1) Y = β_0 _+ β_2_**X**_2 _+ β_1_X_1 _+ ..... β_i_X_i _+ ε

(2) Y = β_0 _+ β_1_X_1 _+ ..... β_i_X_i _+ ε

The difference between the two regression models would evidence the metal exposure effect. The degree of divergence (expressed as TAQ) from the baseline indicates the level of effect of metals on the natural progress of ageing.

Conceptually, the Target Ageing Quotients are defined as the difference between the good/healthy ageing (non-significant metal exposure) and the 'non-healthy' ageing due to excess metal exposure. The baseline 'good/healthy ageing' model may be estimated by reducing the combined level of the metal exposure (ΣTHQ metal) to or below the threshold but keeping all other parameters constant. The β_2_X_2 _component indicates the difference between baseline (estimated theoretical) and observed regression and is intuitively closely related (or can be used to establish) the TAQ.

*H4: The effect of metal exposure on ageing is modified by variables such as genetic and environmental factors*.

Evidence for the modifying effects may be established by comparing regression models of qualitatively different groups with appropriate controls (i.e. fit, content, genetic make up, occupation type, etc). In this case, the β_2_X_2 _component represents the difference between the two groups.

### Data requirements

Secondary data must be analyzed to establish the sampling parameters. Research and national surveys are likely to provide information regarding dietary patterns, may or may not linked to ageing or ageing related degenerative diseases. Primary data include analysis of metal ions, biophysical measures and questionnaires and/or structured interviews for dietary assessment. Biophysical measures and dietary assessment must be paired (both categories are to be measured on each individual participating in the research).

### Variables

Outcome measure for testing H3 and H4 is the ratio of the biological and chronological age. It is calculated by dividing the biological age (expressed as years [14]) by the chronological age so the high > 1 value indicate the 'non-healthy' difference whereas values < 1 indicates delayed ageing.

Methods for estimating biological age typically use batteries of biochemical, physical, mental, and functional parameters that are assumed to vary with age [15]. For example, biological age can be estimated by using measures such as:

▪ forced expiratory volume in 1 second, systolic blood pressure, hematocrit, albumin, and blood urea nitrogen [16]

▪ the concentration of prostacylin in fibroblast, cell membrane viscosity, the electroretinogram, baroreflex regulation of the heart rate, the concentration of lymphocytes, leucocyte densitity and velocity, grip strength, cells of corneal endothelium and the buccal epithelium, neck muscle mobility, and vital capacity [17].

During the pilot study phase, the parsimonious but reliable sets of markers of biological age can be selected from a larger pool by using regression or principal component analysis.

The THQ is calculated by the formula established by the Environmental Protection Agency [10] using equation 3, where EFr is the exposure frequency (days/year); ED_tot _is the exposure duration (year); SFI is the mass of selected dietary ingested (g/day); MCS_inorg _is the concentration of inorganic species in the dietary components (μg/g wet weight); R_f_D: oral reference dose (mg/kg/day); BW_a_: the average adult body weight; AT_n_: averaging time for non-carcinogens (day); and 10^-3^: the unit conversion factor.

(3) $$THQ=\frac{EFr\times ED_{tot}\times SFI\times MCS_{inorg}}{R_{f}D\times BW_{a}\times AT_{n}}\times 10^{-3}$$

### Sample size

The sample is recommended to be drawn from wide cross-section of general population with adequate data in each subgroup of interest. The minimum required sample size depends on effect size and power of the test and is to be established via a pilot study [18,19].

### Proposed approach

Information of 'typical' consumption rate, life expectancies, etc. can be obtained from large scale national surveys (i.e. General Household surveys, National statistics reports). Phase I (pilot) studies should aim to i) select the relevant biomarkers, ii) collect information for estimating the distribution of the level of metal exposure in the population, and iii) measure metal content of selected foodstuff. Food items may be identified via secondary information (large scale food surveys and results on metal content where available) before data collection. Phase II studies focus on gathering empirical evidence to test the relationship between ageing and metal exposure.

### Limitations

Limitations of the proposal arise from the fact that Tolerable Upper Intake Level (UL) have only been established for a small number of metals. In addition, recall of diet may be inaccurate hence we must calculate with a degree of uncertainty. This type of research is limited to longitudinal or cross-sectional observational studies – both require considerable resources but contribute significantly to the body of knowledge.

## Implications of the hypotheses

A particular focus on the types of metal will allow deconvolution of the roles of particular classes of metals (e.g. redox-active and common, redox-active and rare, non-redox active) will allow facilitate determination of the roles of types of metals on ageing.

## Competing interests

The authors declare that they have no competing interests.

## Authors' contributions

DN and AP contributed equally to the development of the hypothesis and preparation of the paper. All authors have read and approved the final manuscript.
